# Improving Implementation: Building Research Capacity in Maternal, Neonatal, and Child Health in Africa

**DOI:** 10.1371/journal.pmed.1000299

**Published:** 2010-07-06

**Authors:** James Whitworth, Nelson K. Sewankambo, Valerie A. Snewin

**Affiliations:** 1 Wellcome Trust, London, United Kingdom; 2 Makerere University, College of Health Sciences, Kampala, Uganda

## Abstract

As part of a series on maternal, neonatal, and child health in sub-Saharan Africa, Valerie Snewin and colleagues discuss the challenges of implementation and research capacity in Africa.

This paper is part of a *PLoS Medicine* series on maternal, neonatal, and child health in Africa.

This *PLoS Medicine* series began by outlining how much we fail mothers, newborns, and children in Africa by not implementing effectively what we know saves lives and improves health [1],[2]. It is clear that countries in Africa are falling behind not only on improving maternal, newborn, and child health but on the Millennium Development Goals 4, 5, and 6 more generally. Why is there such a wide gap between what we know and what we do? While technical knowledge about what could be done is available, actual implementation is neither straightforward nor easy in the often difficult circumstances on the ground. The many competing priorities—along with limited logistic capacity, a lack of political will, and inadequate infrastructure—also constrain the extent to which effective health packages are delivered to those who need them most.

## Implementation Science

It is estimated that between 66% and 85% of Africa's maternal, newborn, and child (under 5 years) deaths could be avoided by implementing current interventions [3],[4]. Therefore, the priority for maternal and child survival is not so much the development of new technologies but solving implementation issues, such as how to scale up and evaluate interventions within complex health systems. Such implementation research should not only focus the attention of policy makers and implementers, but also improve decision making, enhance efficiency, and build understanding of why some programmes work and others do not. But generating the necessary robust evidence is not easy.

First, we do not know how best to scale up interventions effectively. The recent evaluation of UNICEF's Accelerated Child Survival and Development programme in West Africa showed that, while vertical preventive implementation did improve coverage, there was no acceleration in child survival [5]. Similar rigorous evaluations of other existing large-scale implementation programmes such as PEPFAR, the Global Fund to Fight HIV/AIDS, Tuberculosis and Malaria, and GAVI would help accelerate progress towards better implementation [6].

However, the evaluation of complex interventions is itself problematic and more work needs to be done on the development of robust and generally accepted methods for such evaluations [7],[8]. While randomised controlled trials are considered the gold standard for evaluating interventions, there is little consensus on when these should be applied for evaluating complex interventions, or on what other methods are appropriate and in what circumstances.

## Engagement of Southern Voices and Institutions

It is clear that there is a need to broaden the base for health research in low- and middle-income countries, especially for implementation research [9]. But how can sub-Saharan African countries strengthen their often weak health systems while at the same time increase their own capacity to do research to improve the health of not only mothers, newborns, and children, but of their entire population? A first step would be to listen to the voices of those grappling with the issues on the ground. Too often well-meaning initiatives are developed in Washington, Geneva, or London without incorporating the views of African scientists, policy makers, and civil society. Of course, the global community, including the H8 group of health organisations and the G20 group of major advanced and emerging economies, has a major role to play in realising the aims of building capacity in Africa, but this needs to be done while taking into account the voices of those on the ground. Until recently it has been difficult to obtain an authoritative voice that represents a wide spectrum of African scientists. But things are changing, and the recently established Initiative to Strengthen Health Research Capacity in Africa (ISHReCA; http://ishreca.tropika.net/) aims to serve as a forum for African scientists to collate ideas on capacity building and to speak with a collective voice. ISHReCA has identified a series of key requirements for strengthening health research capacity in Africa, focused around the need to improve the research environment, and for supporting both individuals and institutions [10],[11]. This effort is relevant across the whole spectrum of scientific research, because, while there is an imperative to implement what we already know, there is still a need to develop better interventions and delivery strategies.

## Improving the Research Environment

In many African countries legislation needs to be modernised to support the conduct of research, to exchange materials and data, and to protect intellectual property rights. African governments also need to make greater efforts to support research through strategic planning, strengthened research governance, and increased funding. Equally, African governments need to develop strategic plans for increasing and supporting human resources for research for health in parallel with the requirements of the programme implementation work force. All this is unlikely to happen without increased engagement by scientists and advocates to promote science within African societies and to demonstrate the benefits that investment in research can contribute to health development and wealth creation. Strong, sustained advocacy is required to encourage policy makers to ensure that research is supported by increased financial and political support [12]. National Academies of Science (strengthened through the African Science Academy Development Initiative and Royal Society–Pfizer African Academies Programme), the African Academy of Sciences, and the African Union could all be credible advocates to promote the cause of science. National governments should also increase their research funding to match commitments, for example, by allocating at least 2% of health ministry budgets to research [13]. Competitive national grant schemes with merit-based peer-reviewed assessments are required. An example of such a scheme is in Uganda, where the government together with the World Bank has funded the Millennium Science Initiative. Calls for proposals are issued regularly through the press. Applicants submit proposals, which are reviewed by a team of both Ugandan and international scientists, and awards are made on a competitive basis.

## Supporting Individuals

There is an urgent need to build the next generation of African scientists. Schoolchildren need to be instilled with excitement about science through their teachers and curricula, otherwise they are unlikely to choose to study science subjects at university. Universities need to promote and support research as well as training and service, so that undergraduates are exposed to research and taught by researchers throughout their courses, hopefully leading them to view research as an attractive career option. However, to facilitate this credible career paths must be created which offer opportunities at every level. Attractive packages with competitive salaries, career posts, and opportunities for training and travel are important, as is special attention to the recruitment of women. More programmes are needed that promote good mentoring and empower junior scientists. For example, at Makerere University in Kampala, clinical scholarship positions have been created to attract, mentor, and retain junior researchers, and there is a fast-track pathway for promotion based on research productivity. Senior scientists themselves need to be identified who will act as research leaders and role models. Such research group leaders need to be supported with secure funding, for example through endowed positions, to enable them to help and mentor young researchers throughout their careers. Opportunities for funding need to be diversified beyond the usual international foundations and agencies, to include national governments, private donations, local charities, and corporations.

## Supporting Institutions

The infrastructural base for research at most institutions in Africa needs much improvement. African governments need to contribute more to providing basic facilities, providing a foundation upon which external agencies can build. Funding agencies and donors need to work together to ensure that the true costs of research are provided for, to include such overhead as upgrading facilities and support services such as information technology, library services, ethical oversight, laboratory support, and financial and research management. Clearly not all universities or higher education facilities can be supported in this way, and priority should be given to those research institutes and universities that have the potential to flourish. Health research funders and development agencies need to ensure that there is greater harmonisation between themselves and increased alignment with national health priorities, to avoid unnecessary duplication of effort and divergence of aims [14]. South Africa, while not a typical sub-Saharan country, has shown a promising way forward. The University of Cape Town Health Sciences Faculty has eight well-funded research chairs, each provided with two research assistants and research funds. For every PhD and Masters degree successfully completed, and every publication, universities in South Africa receive funding from government. This helps to incentivise universities to train research students and for researchers to publish.

## Developing Networks

Just as with intervention research, there is an urgent need to evaluate initiatives that aim to strengthen research capacity by using robust and generalisable methods and to share learning from them. Relatively few examples of this process exist, and the literature is sparse ([10] and Box 1). The Wellcome Trust is supporting a thorough evaluation of the recently launched African Institutions Initiative [15].

**Box 1. Initiatives and Networks for Research Capacity Strengthening in Africa**

- Healthy Newborn Network: http://www.healthynewbornnetwork.org
- African Health Research Forum and University Science, Humanities and Engineering Partnerships in Africa (USHEPiA): http://web.uct.ac.za/misc/iapo/ushepia/bg.htm
- European and Developing Countries Clinical Trials Partnerships (EDCTP) Networks of Excellence: http://www.edctp.org/
- European Union funded Network for the Co-ordination and Advancement of sub-Saharan Africa-EU Science and Technology Cooperation (CAAST-Net): http://www.caast-net.org
- Health Research Capacity Strengthening initiative (HRCS) a partnership between UK Department for International Development (DFID), International Development Research Centre (IDRC), Canada and the Wellcome Trust: http://www.wellcome.ac.uk/hrcs
- INDEPTH Network (International Network of field sites with continuous Demographic Evaluation of Populations and Their Health in developing countries): http://www.indepth-network.org/
- Initiative to Strengthen Health Research Capacity in Africa: http://ishreca.tropika.net/
- Malaria IPTi network (funded by the Bill & Melinda Gates Foundation, WHO, and UNICEF): http://www.ipti-malaria.org
- Medical Research Council (UK) and DFID African Research Leader scheme: http://www.mrc.ac.uk/Fundingopportunities/Calls/AfricanResearchLeader/MRC006652
- Neglected Tropical Diseases Fellowship Scheme (supported by a consortium of European foundations): http://www.ntd-africa.net
- Netherlands African Partnership for Capacity Development and Clinical Interventions against Poverty-related Diseases; Netherlands Organisation for Scientific Research: http://www.nwo.nl/naccap
- Leverhulme Royal Society Africa Awards: http://royalsociety.org/Leverhulme-Royal-Society-Africa-Awards/
- Wellcome Trust African Institutions Initiative: http://www.wellcome.ac.uk/aii

Partnerships and networks should be encouraged to promote North–South and South–South interaction. Too often partnerships are developed between a northern university research powerhouse and a much smaller, less research-active, African university. This imbalance is unlikely to lead to serious sustainable capacity development in the South. Equitable partnerships built upon mutual trust must be encouraged [16]. Increased support for South–South networks is also desirable so that established universities can assist the development of emerging neighbouring institutions. Funding agencies, including national governments, can promote collaborative networks to build lasting change.

## Conclusion

The high levels of maternal, newborn, and childhood mortality and morbidity in Africa are cause for an urgent response to implementing interventions. Strong health research systems and research programmes that address bottlenecks to upscaling effective interventions should be developed without delay. This effort requires substantial and rapid investment in the support of African scientists, institutions, and systems that will focus on solutions to African problems.

## Abbreviations

GAVI: Global Alliance for Vaccines and Immunisation
PEPFAR: US President's Emergency Plan for AIDS Relief
UNICEF: United Nations International Children's Fund
